# Quorum sensing signals improve the power performance and chlortetracycline degradation efficiency of mixed-culture electroactive biofilms

**DOI:** 10.1016/j.isci.2022.104299

**Published:** 2022-04-26

**Authors:** Xiao-Long Cheng, Qiang Xu, Jia-Dong Sun, Chun-Rui Li, Qian-Wen Yang, Biao Li, Xue-Ying Zhang, Jun Zhou, Xiao-Yu Yong

**Affiliations:** 1 College of Biotechnology and Pharmaceutical Engineering, Bioenergy Research Institute Nanjing Tech University, Nanjing 211816, China; 2 Department of Environmental Engineering, Technical University of Denmark, DK 2800, Lyngby, Denmark; 3 School of Environmental Science and Engineering, Nanjing Tech University, Nanjing 211816, China

**Keywords:** Chemistry, Electrochemistry, Electrochemical energy conversion, Earth sciences, Environmental science, Energy resources, Biological sciences, Microbiology, Biotechnology, Biological waste treatment

## Abstract

Electroactive biofilms (EABs) play an important role in bioelectrochemical systems due to their abilities to generate electrons and perform extracellular electron transfer (EET). Here, we investigated the effects of quorum sensing (QS) signals on power output, chlortetracycline degradation, and structure of EABs in MFCs treating antibiotic wastewater. The voltage output of MFCs with C4-HSL and PQS increased by 21.57% and 13.73%, respectively, compared with that without QS signals. The chlortetracycline degradation efficiency in closed-circuit MFCs with C4-HSL and PQS increased by 56.53% and 50.04%, respectively, which resulted from the thicker biofilms, higher biomass, and stronger activities. Additionally, QS signals induced the heterogeneous distribution of EPS for a balance between self-protection and EET under environmental pressure. *Geobacter* prevailed by the addition of QS signals to resist high chlortetracycline concentration. Our results provided a broader understanding on regulating EABs within electrode interface to improve their performance for environmental remediation and clean energy development.

## Highlights

• The voltage output of MFCs was enhanced with the addition of QS signals
• QS signals increased the bioelectrochemical degradation efficiency of CTC
• EABs exhibited heterogeneity in composition and interaction by the QS signals
• QS signals induced a balance between self-protection and EET of EABs

## Graphical abstract

**Figure:**
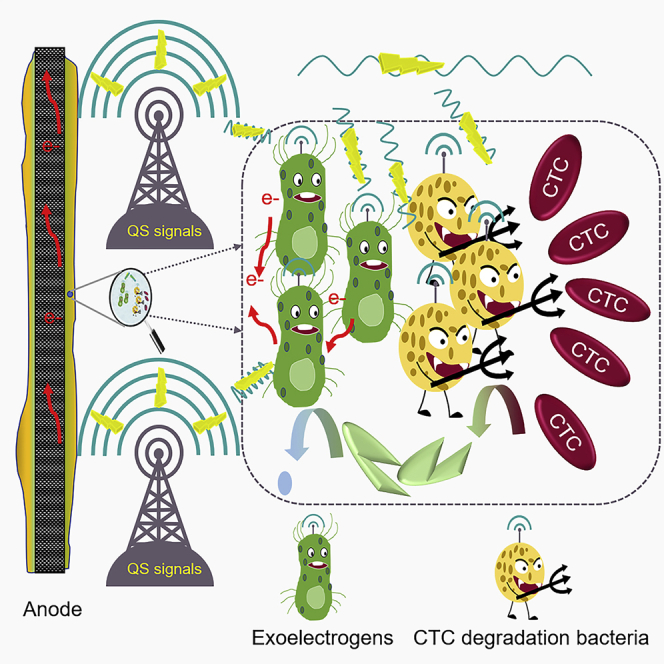

## Introduction

As a broad-spectrum antibiotic, chlortetracycline (CTC) is widely used in animal husbandry due to its strong antibacterial activity against a variety of pathogens. However, the discharge of inadequately treated CTC wastewater into the environment poses a serious threat to human health (Chen et al., 2020). Generally, wastewater containing CTC can be treated by the processes based on membrane filtration (Monzon et al., 2016), photocatalysis (Zhang et al., 2017b), adsorption (Zhang et al., 2020), and so on. However, certain defects, such as susceptibility to environmental impacts, high costs, and secondary pollution block their ways for wide application. On the other side, as an environmentally friendly bioelectrochemical technology to convert chemical energy stored in wastewater into clean electricity, microbial fuel cells (MFCs) provide an alternative to treat wastewater/pollutants, which received much attention in environmental remediation in the past decade (Yong et al., 2014).

A large number of studies have reported that the anode biofilms of MFCs can degrade antibiotic contained in wastewater containing antibiotics, such as sulfamonomethoxine (Jiang et al., 2020), salinomycin (Cheng et al., 2020), and sulfamethoxazole (Wang et al., 2016), since it has the advantages of lower cost, better treatment efficiency, as well as the simultaneous generation of clean energy. When treating wastewater containing pollutants, exoelectrogens require higher tolerance and electron transfer ability to maintain their metabolism. As previously reported, mixed bacteria culture has a stronger capability than pure bacteria in resisting the stimulation of external environmental pressure and toxic pollutants. Specifically, our previous reports demonstrated that fermentative bacteria degrade complex organics into small molecules that can be further utilized by exoelectrogens in the anode chambers of MFCs (Li et al., 2021). This synergetic effect is the basis for simultaneous degradation of organic pollutions and generation of clean power by mixed electroactive biofilms (EABs) in MFCs (Li et al., 2019a, 2021).

Similar to general biofilms, the formation of EABs is mainly divided into four stages: 1) Attachment of microbial cells on electrode; 2) Secretion of extracellular polymeric substrates (EPS) and biofilm formation; 3) Cells growth and biofilm maturation; 4) Cells detachment and biofilm regeneration (Czerwinska-Glowka and Krukiewicz, 2020). The formation of EABs was the basis for the biological function of exoelectrogens in natural environment. For example, exoelectrogens could exchange electrons between EABs and electrode during metabolism via extracellular electron transfer (EET) (Hou et al., 2020). Besides, EPS wrapped around microorganisms was wildly studied due to its spatial distances and compact architecture. Accordingly, biofilm dynamics is regulated by the intercellular information exchange, known as quorum sensing (QS), which relies on the production, secretion, and perception of QS signals (Tan et al., 2020).

Based on the types of QS signals, QS systems are mainly divided into three main categories namely acyl homoserine lactones (AHLs), auto-inducible peptides (AIP), and auto-inducible factors. And beyond that, others include quinolone (PQS) and inter/intraspecies diffusion signals (Du et al., 2021; Waters and Bassler, 2005). It is known that the QS regulation system in Gram-negative bacteria was based on LuxI/LuxR system mediated by AHLs (Schuster et al., 2013). At the present stage, the widely reported exoelectrogens were almost Gram-negative bacteria such as Geobacter and Shewanella, which were believed to be regulated under AHLs. In addition, rather than directly participating in QS, PQS acted as a signal agent between *las* and *rhl* systems and played a different role with AHLs in QS system (Brindhadevi et al., 2020). Despite the differences in QS systems among bacterial species, however, they all have the essential functions that regulate a variety of behaviors such as biofilm formation, granular sludge formation, and membrane biofouling due to the close cell-to-cell communication (Du et al., 2021; Mukherjee and Bassler, 2019). C4-HSL, which is regarded as a representative AHLs, was reported to play an important role in the communication among/between different microorganisms (Chen et al., 2017b; Schuster et al., 2013). Besides, Sun reported that C4-HSL was associated with biofilm formation through affecting EPS production (Sun et al., 2018). Similarly, PQS was also found to promote the growth of biofilm since the addition of PQS increased the content of protein in EPS and biomass by upregulating the *rhlRⅠ* QS system, which was favorable for biofilm maturation (Zhao et al., 2022).

Additionally, numerous studies have found that QS signals play an important role in regulating the removal of pollutants in various species of microorganisms and communities (Kang and Park, 2010; Zhang et al., 2015). It is also reported that the degradation of nicotine was almost 100% with the addition of QS signals while that was less than 20% in quorum-quenching (QS was interrupted by some specific measures) reactors (Zhang et al., 2015). As previously reported, QS signals could accelerate the biofilm formation and enhance its resistance to harsh external environment. Nevertheless, to the best of our knowledge, few studies focused on the synergistic effects to active response for a good balance between self-protection and EET under environmental pressure among mixed organisms. Therefore, it is necessary to understand the role of QS signals in the formation of EABs and the relationship between power generation and pollutants degradation within electrode interface.

In this study, two kinds of QS signals (N-butyl homoserine lactones, C4-HSL; quinolone, PQS) were supplied to the anode chambers of MFCs to 1) clarify their influence on the electrochemical performance; 2) illustrate the regulation of QS signals on the structure, composition, and activity of EABs; 3) and establish a QS-regulated bioelectrochemical strategy for efficient degradation of CTC. Results showed that QS signals shortened the maturation period of EABs by promoting their enrichment, increasing EPS secretion, and enhancing the EET by decreasing the charge transfer resistance. Consequently, the shaped EABs promoted the performance of CTC degradation and electricity generation simultaneously. This study provides an alternative strategy to modulate the electrochemical performance of MFCs and broaden its potential for application in environmental remediation.

## Results and discussion

### QS signals accelerated the start-up and increased the power output of MFCs

The influence of QS signals on the electrochemical performance of MFCs was first analyzed based on the variation of voltage output during the enrichment stage (without the addition of CTC). The voltage output of the MFCs with the addition of C4-HSL and PQS increased significantly in the third cycle, which stabilized after the fifth cycle and maintained with the maximum values around 0.62 and 0.58 V, respectively (Figure S1). However, the voltage output of the MFC without QS signals increased slowly in the early stage and the maximum voltage of each cycle was significantly lower than that of the C4-HSL and PQS groups. Specifically, the voltage output of the Blank group stabled at 0.51 V in the sixth cycle, which was 17.74% and 12.07% lower than that of the C4-HSL and PQS groups, respectively. In addition, the voltage output of abiotic groups was negligible compared with that of the biotic groups supplemented with C4-HSL and PQS (Figure S2). Therefore, we concluded that the voltage output was attributed to the metabolism of exoelectrogens on EABs and the performance difference of these MFCs was due to the impact of C4-HSL and PQS on microorganism.

**Figure 1:**
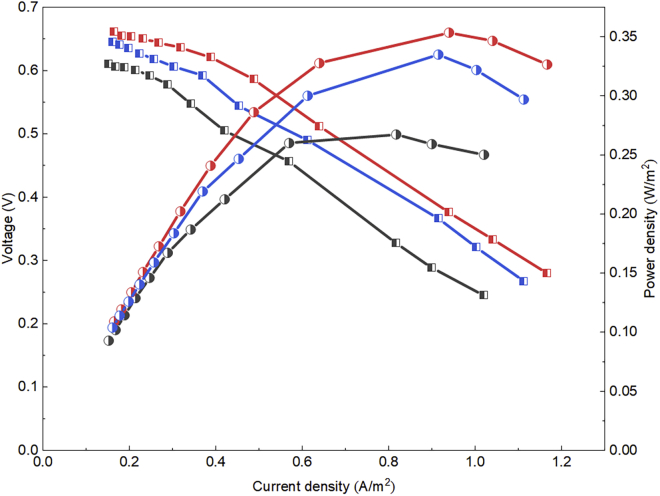
The polarization curve (square symbol) and power density (circle symbol) of the three MFC groups (Blank: black, C4-HSL: red, and PQS: blue) during the enrichment stage of EABs

Interestingly, QS signals also accelerated the start-up of the MFCs. To be more specific, the start-up time of C4-HSL and PQS groups was both 240.35 h, which was 19.94% shorter than that of the Blank group (300.22 h). It is well known that the exoelectrogens attached on anode surface breaked the electron donors intracellular through anaerobic oxidation and then transferred the generated electrons to electrode to form a voltage output (Guo et al., 2020). The faster started-up and stabilization of the MFCs with C4-HSL and PQS indicated that QS signals reinforced the attachment of exoelectrogens to form EABs on anodes, which promoted EET and increased the maximum output voltage by 21.57% and 13.73%, respectively, compared to that of the Blank group. Meanwhile, the maximum power density (0.35 and 0.33 W/m^2^) also increased by 34.62% and 26.92% with the addition of C4-HSL and PQS, respectively (Figure 1).

**Figure 2:**
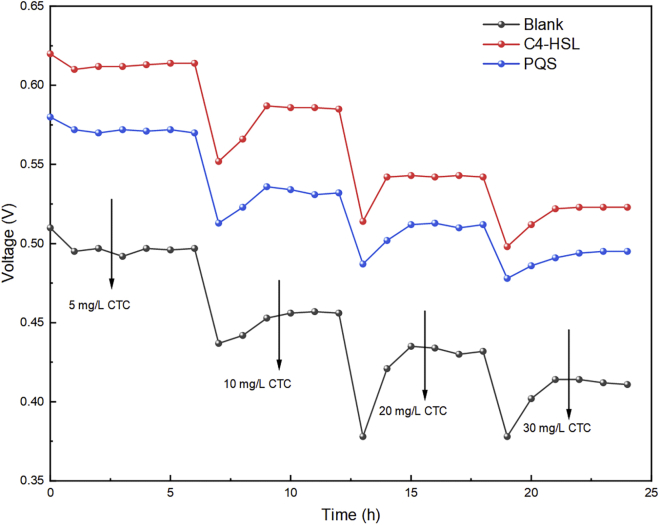
Voltage output of the three MFCs groups (Blank, C4-HSL, and PQS) during CTC (5–30 mg/L) domestication stage

Furthermore, different concentrations of CTC were added to anode chambers to acclimate the EABs for simultaneously degrading CTC and generating electricity after enrichment. Based on the effect of CTC on the activity of EABs, the inhibition of CTC on the voltage output in four batches was calculated according to the equation (Text. S4). The maximum voltage of the three MFCs groups stabilized after nearly 600 h’ enrichment, which possibly indicated that the formation of EABs matured on the anode at this stage (Figure S1). When CTC concentration increased from 5 to 30 mg/L, the voltage outputs were all suppressed to different degrees in each MFCs groups (Figure 2) attributed to the toxicity of CTC. The maximum voltage outputs corresponding to the C4-HSL, PQS, and Blank groups at 30 mg/L CTC were 0.52, 0.50, and 0.41 V, which were respectively 15.65%, 14.66%, and 17.20% lower than that in enrichment stage (Table 1). The inhibition rates of voltage output in C4-HSL and PQS groups were apparently lower than that in Blank group, indicating that QS signals exerted a beneficial effect on the tolerance of EABs with the increased acclimation duration. Changes in external environment are a significant reason for the changes of physiological activity of microorganism (Li et al., 2019a). Interestingly, the different inhibition rates between the C4-HSL group and PQS group under CTC domestication stage were nonnegligible, which could be due to the different effects of two QS signals on the regulation of metabolic pathways (Mukherjee and Bassler, 2019). At this time, the microbial community needed to adjust their structure and abundance, as well as regulating the gene expression, to response and resist the toxicity of CTC to make their metabolism consistent with the physiological needs for using CTC as energy supplier and electron donor (Ma et al., 2021).

**Table 1:** The inhibition rate of different concentrations of CTC on the voltage output of MFCs with the addition of QS signals

Concentration(mg/L)	Inhibition (%)		
	C4-HSL	PQS	Blank
5	0.97 ± 0.13	1.72 ± 0.28	0.60 ± 0.09
10	5.32 ± 0.98	7.59 ± 1.02	8.60 ± 1.47
20	12.42 ± 1.57	11.55 ± 1.23	13.01 ± 1.34
30	15.65 ± 1.92	14.66 ± 2.24	17.20 ± 1.56

### QS signals enhanced the bioelectrochemical degradation of CTC

**Figure 3:**
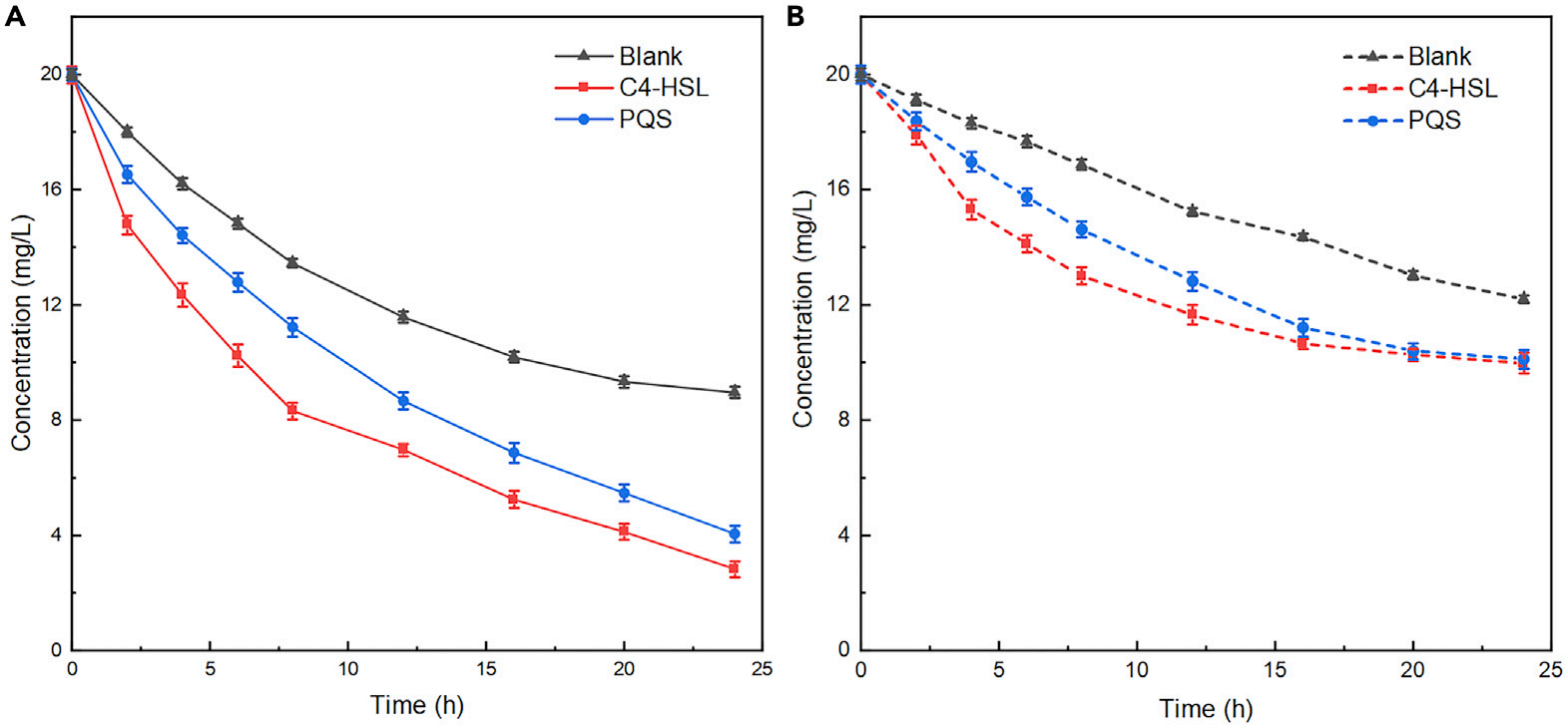
Degradation-time diagram of CTC in three groups of MFC (Blank, C4-HSL, and PQS) with different-acclimated EABs/biofilm: a) electrochemical degradation under closed circuit; b) anaerobic fermentative degradation under open circuit

CTC was degraded in anode chambers of all three MFCs groups (without glucose), indicating that the mixed microorganisms embedded in EABs can use CTC as the sole carbon source for metabolism and power generation, which is consistent with the above results (Figure 2). At the same time, abiotic control experiment (without strains) confirmed that the removal of CTC was not the result of adsorption by carbon felts (data not shown). It is worth noting that the CTC degradation efficiency of the C4-HSL and PQS groups was 85.85% and 82.85%, respectively, which were 1.56 and 1.50 times that of Blank group (55.20%) (Figure 3A). Similar to the closed-circuit treatments, CTC was also removed in the C4-HSL, PQS, and Blank group within 24 h under the open-circuit anaerobic fermentative controls, with the degradation rates of 50.10%, 49.40%, and 39.05%, respectively. Obviously, however, the degradation of CTC could be improved significantly by supplying QS signals no matter that in the electrochemical degradation under closed circuit or anaerobic fermentative degradation under open circuit. More importantly, we found that the lower concentration of CTC was observed in the MFCs than that of the fermentative runs (Figures 3A and 3B), which also showed the superiority of electrochemical treatment of pollutants (Li et al., 2019a). In previous report, CTC was converted into lower toxic intermediates in the biosystem (Zhou et al., 2021). Although the intermediates of CTC were not detected in this study, we could speculate that CTC was degraded into lower toxic intermediates with low molecular weight, which was favorable for the survival of microorganisms. The same conclusion was reported in our previous study (Li et al., 2019b).

In addition, the rate constants of CTC degradation in C4-HSL and PQS groups were 2.19 and 1.86 times (under electrochemical degradation) and 1.35 and 1.42 times (under fermentative degradation) of the Blank group, respectively (Table S3, Figure S3), which were calculated by the degradation kinetic equation. As discussed above, the rate constants of CTC degradation in MFCs were all superior than that in the fermentative systems. This might be related to the synergetic effect between the mixed bacteria species in the EABs, which was discussed as following.

### QS signals regulated the activity of the EABs

It has been reported that the three-dimensional EABs on anode improved the bacterial tolerance to toxic pollutants and thus the performance in removing organic pollutants also been significantly enhanced (Li et al*.*, 2021). In the present study, the electrochemical performance of each MFCs was also characterized after the enrichment of EABs.

Voltammetry is a dynamic technique which is sensitive to both electron transfer kinetics and major mass transfer at the electrochemical interface. As the sweep speed increased, the difference between the oxidation and reduction peak potentials as well as the catalytic currents of the three MFCs groups increased, indicating the presence of redox substances at the interface between the anode surface and the solution formed parts of EET of the EABs. There was no obvious difference between the oxidation peak and reduction peak potential of the three MFCs groups under the same scanning speed (Figure S4), which indicated that the addition of exogenous QS signals neither induced the EABs to produce new redox substances nor acted as electron transfer shutters. Nevertheless, the catalytic currents of the C4-HSL and PQS groups were 21.35 and 19.98 mA at a sweep rate of 0.05 v/s, respectively, which were 50.04%, and 40.40% higher than those of the Blank group (14.23 mA) (Figure S4). This demonstrated that the activity of the intrinsic redox substances was significantly increased due to the addition of QS signals (Liu et al., 2021).

In addition, the electron migration rate constants of the C4-HSL and PQS groups were calculated to be 0.030 ± 0.0068 and 0.027 ± 0.0019, respectively, which were 1.58 and 1.42 times than those of the Blank group (0.019 ± 0.0021).

**Figure 4:**
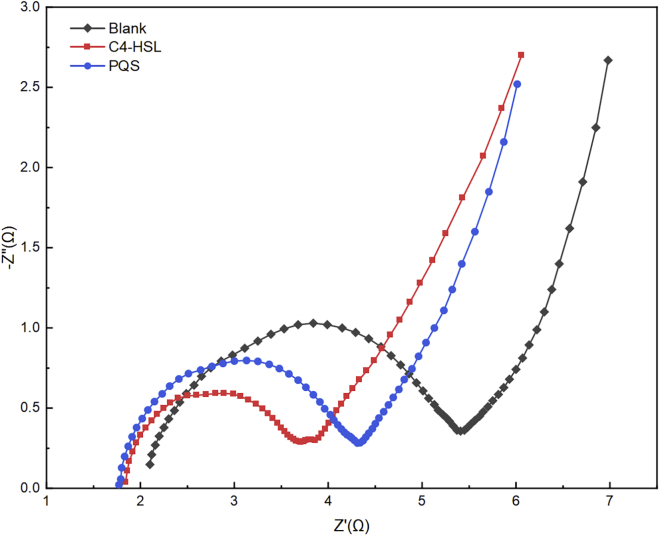
The Nyquist curve of the three MFC groups at scan rate of 10 mV, in the frequency range of 10 kHz–100 mHz

The enhanced electron migration rate was a significant feature of the decreased resistance of the EABs. Electrochemical impedance (EIS) was employed to assess the conductivity and the electron diffusion ability of the EABs in the MFCs with QS signals. Generally, the charge transfer resistance (Rct) can be obtained from the diameter of the semicircle in the Nyquist curve represents (Liu et al., 2020). As shown in Figure 4, the Rct of the three MFCs groups decreased from 3.41 Ω (Blank) to 1.93 Ω (C4-HSL) and 2.26 Ω (PQS), highlighting the effectiveness of QS signals in enhancing electrons transfer between anode surface and EABs. On the other hand, the solution resistance (Rs) in all groups had no obvious difference. Considering Rct occurs between electrode and EABs, this observation may be involved in the composition of EPS wrapping around the cells (discussed below).

### Morphological characteristics of the EABs

The cell morphology embedded in EABs on the anode surface of the three MFCs groups was detected by SEM (Figure S5). The amount of bacteria cells attached on the anode surface at the initial stage of biofilm enrichment (first cycle) was relatively low. In the middle of the enrichment period (fourth cycle), however, EABs exhibited obvious three-dimensional and complex structures compared with that of the initial stage, which was in accordance with the increased voltage output. In the fifth cycle (and the sixth cycle of the Blank group), the voltage output of each MFCs group was stabilized (Figure S1), indicating that the EABs matured at this time after enrichment. Moreover, in the later stage of enrichment, dense EABs layers were formed with spherical and rod-shaped strains uniformed distributed on the anode surface.

### EPS composition and biomass regulated by QS signals

**Figure 5:**
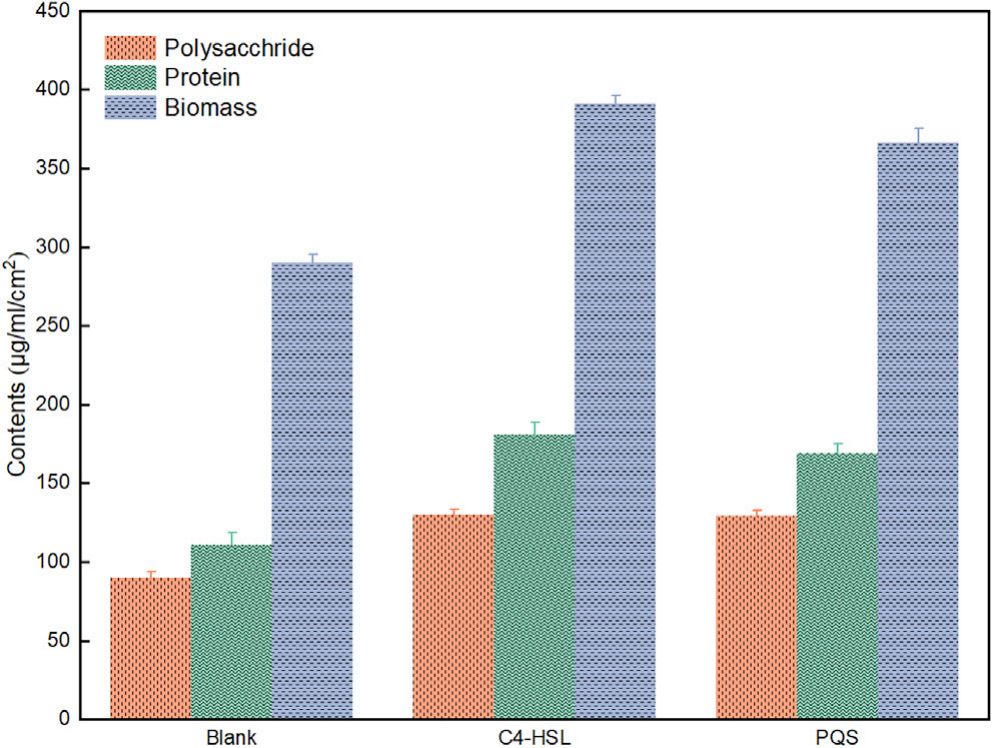
Biomass; protein and polysaccharide of EPS in the three MFC groups during enrichment stage of EABs

The mixed bacteria attached on anodes surface were wrapped with uneven thickness EPS and gradually formed EABs during enrichment. Cells in EABs have been thought to transfer electrons between redox radical in the EPS and insoluble electrode (Gao et al., 2019). Here, the biomass density increased from 290.32 (Blank) to 390.98 (C4-HSL) and 366.33 μg/mL/cm^2^ (PQS) (Figure 5), which was in good according to the results of SEM It could be seen that the two QS signals showed various impact on the evolution of EABs, which could be revealed from the voltage output, CTC degradation efficiency, EIS, and biomass density. The differences could be resulted from the direct effect by C4-HSL and/or indirect effect by PQS in the QS system (Brindhadevi et al., 2020; Schuster et al., 2013).

The spatial distances and compact architecture enabled EPS nonnegligible effects in self-protective property and EET. Electrons generated by exoelectrogens cannot easily cross the dense EPS layer to electrode directly (Yang et al., 2019). Fortunately, several electrochemical active substrates embedded in EPS, such as extracellular redox proteins, were certificated to transport electrons between the outer membrane of exoelectrogens and electrodes to facilitate EET process (Xiao et al., 2017). Therefore, the protein content in EPS has a great impact on EET rate. As shown in Figure 5B, the protein content of EPS in the C4-HSL and PQS groups was 180.56 and 169.13 μg/mL/cm^2^, respectively, which was significantly elevated compared to that of the Blank group (110.68 μg/mL/cm^2^). The enhancement in polysaccharide content was also obvious, which increased from 90.10 (Blank) to 130.08 (C4-HSL) and 129.59 μg/mL/cm^2^ (PQS). The rise in polysaccharides content was closely related to the response of biofilms to the changes of external environment, while proteins contain a variety of redox functional groups that facilitated the transfer of electrons outside the cell (Hou et al., 2020).

Surprisedly, QS signals have less effect on the change of polysaccharide content but more on that of protein content. It was speculated that the effects of QS signals have reached the threshold for microbial attachment and response to external stress after the maturation of biofilm; however, their own physical and chemical properties had different effects on electron transfer, resulting in significant differences in protein content in EPS. The higher efficient EET in the EPS of C4-HSL group was obtained compared with other groups, due to the fact that proteins in EPS contained multiple types of redox molecules, while polysaccharides were widely regarded as the insulative substrates (Li et al., 2021; Xiao et al., 2017). When EABs were under the regulation of QS signals, it was favorable for inducing higher content of protein and polysaccharide in this work, which was beneficial to format a heteropical EPS (Li et al., 2021). Moreover, as previous reports, the maximum EET generated by EABs was positively related to the protein content in EPS (Yang et al., 2019). The similar result (the negative relevance between Rct and protein content in the EPS) was obtained in Figure S6 (R^2^ = 0.99), which agreed with the previous report that the increase in the content of conductive protein accelerates the rate of EET in the EABs.

**Figure 6:**
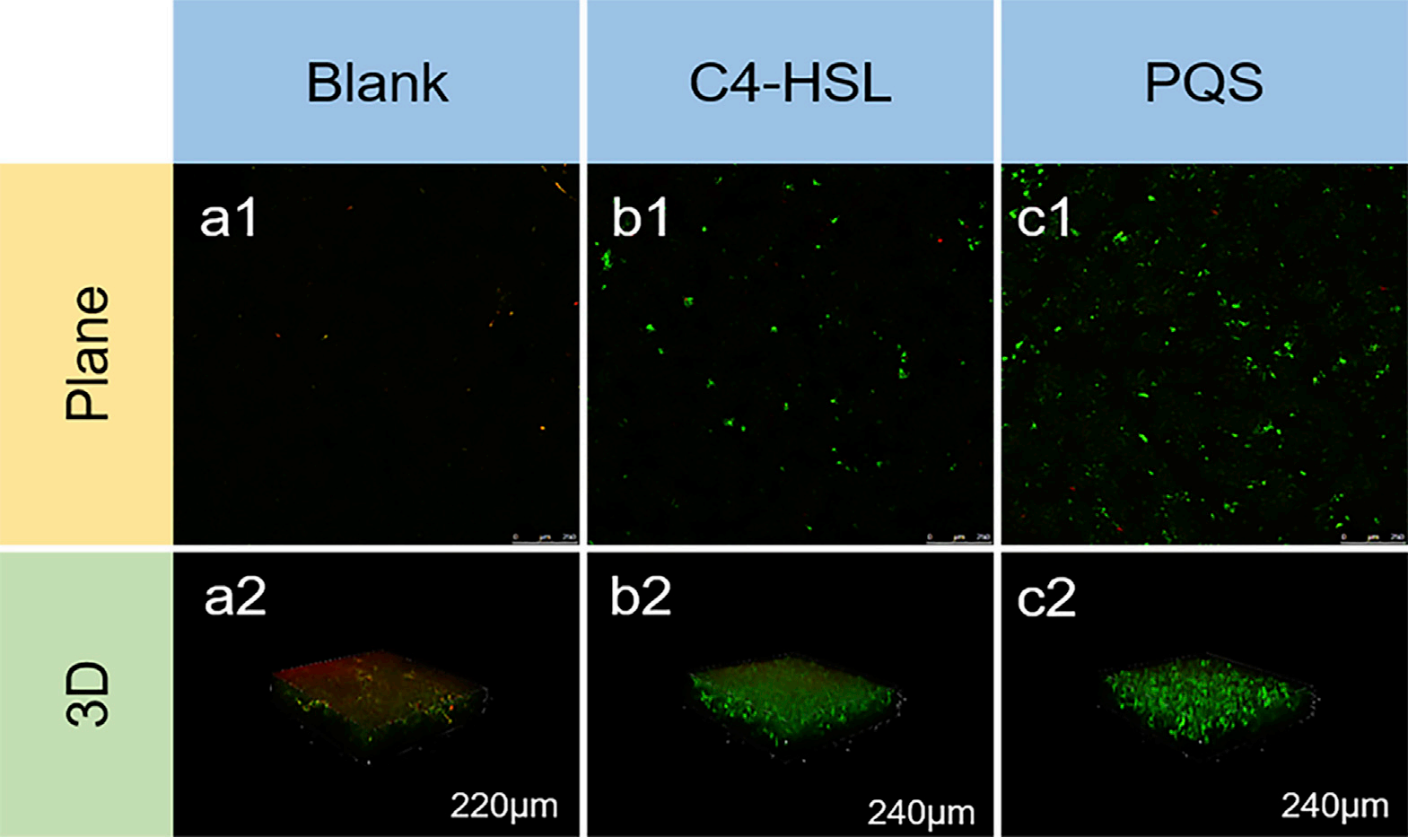
Biofilms of three MFC groups (a: Blank; b: C4-HSL; c: PQS) analyzed by CLSM including plane and 3D

Additionally, the viability of the EABs of the three MFCs groups was analyzed with CLSM, which demonstrated that cells activity in C4-HSL and PQS groups maintained well after a period of stable operation (Figure 6). This was due to the fact that QS signals accelerated the metabolism and communication among species in EABs, which in turn produced a thicker biofilm layer and higher biomass. This played an important role in stabilizing the structure and function of the EABs. As the thickness of the biofilm increasing, the inner cells have a restricted access to nutrients, and their cellular activity also relatively weakened (Sun et al., 2015). In this study, however, EABs under the regulation of QS signals contained fewer inactive cells compared with that of the Blank group, which elaborated that the higher viability of EABs could be achieved with the addition of QS signals. It is well known that the live cells are accountable for electron production (Yang et al*.*, 2019), and the inactive cells might be a reason for the declined viability and blocked EET, thus diminished the EABs’ performance.

### Dynamic shifts in the microbial community after enrichment and CTC domestication

**Figure 7:**
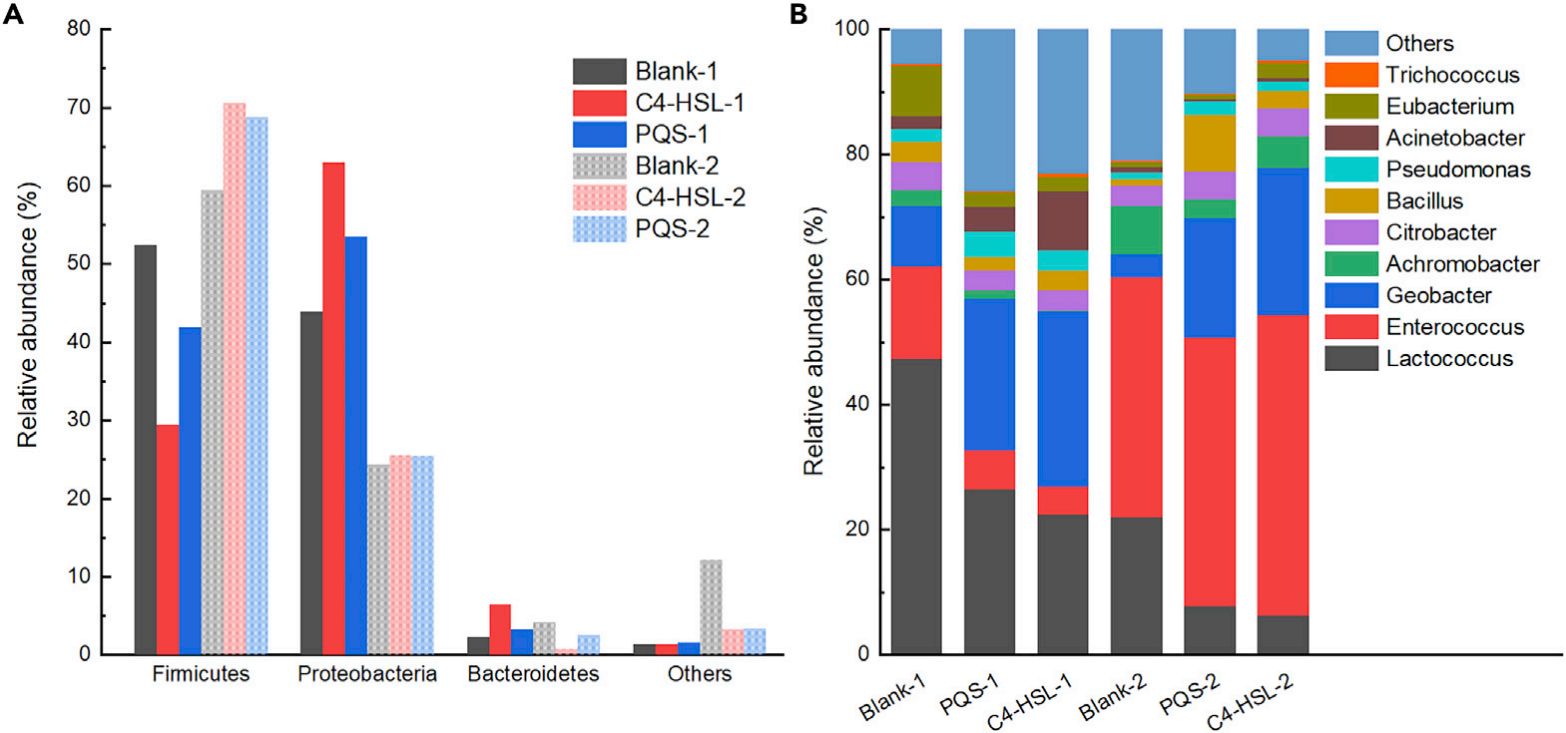
The microbial community composition of the three anode biofilms in different periods (1: enrichment stage; 2: domestication stage) at phylum level (a) and genus level (b)

The microbial diversity of the EABs was analyzed at the end of the biofilm enrichment and CTC domestication to reveal the effects of QS signals on the composition of EABs. At the phylum level (Figure 7A), the EABs of the three MFCs groups in the biofilm enrichment and domestication stages are mainly composed of Firmicutes, Bacteroidetes, and Proteobacteria. It has been reported that Firmicutes and Proteobacteria are main exoelectrogens, which possessed electron-transfer pathways that electrically connected intracellular oxidative catabolic reactions to extracellular terminal electron acceptors (Chen et al., 2017a). However, during the biofilm enrichment stage, the addition of QS signals increased the proportion of Proteobacteria from 43.93% (Blank) to 62.93% (C4-HSL) and 53.44% (PQS), but decreased the percentage of Firmicutes from 52.36% (Blank) to 29.38% (C4-HSL) and 41.81% (PQS). QS mediated by C4-HSL and PQS was present in Gram-negative bacteria (Waters and Bassler, 2005), which could explain why the percentage of Proteobacteria (Gram-negative bacteria) and Firmicutes (Gram-positive bacteria) showed the opposite trend.

The abundance of Firmicutes had the same upward trend in all three groups (59.36% of Blank; 70.54% of C4-HSL; and 68.76% of PQS) after CTC domestication, and yet the remarkable decline occurred in Proteobacteria proportion (24.30% of Blank; 25.53% of C4-HSL; 25.40% of PQS). Studies have reported that Firmicutes have better properties against CTC (Zhang et al., 2017a), which could be the reason for the increased proportion after CTC domestication. In addition, CTC significantly decreased the voltage output of the three MFCs groups. We speculated that Firmicutes may act as CTC degradation biocatalyst, while Proteobacteria had a greater role on power output. As shown in this study, QS signals have different effects on EABs, namely, increasing the percentage of the exoelectrogens during the enrichment while increasing the CTC degradative species after CTC domestication. Intense stimulation of CTC may strengthen the degradation ability of Firmicutes, which moderated the adverse effects on the viability of Proteobacteria, and thus maintained the bioelectrochemical activity of the EABs.

Additionally, at the genus level (Figure 7B), the microbial composition of the EABs in all MFCs groups was mainly *Lactococcus*, *Geobacter*, and *Enterococcus*. *Lactococcus* was reported to belong to fermentative strain (Cavanagh et al., 2015), which accounted for 47.31% in the Blank group, and further decreased to 22.4% in the C4-HSL group and 26.7% in the PQS group during the biofilm enrichment stage. The same declining trend of *Lactococcus* was observed after CTC domestication, in which the relative abundance of *Lactococcus* decreased from 22.01% (Blank) to 6.33% (C4-HSL) and 7.69% (PQS), respectively. In contrast, the percentage of *Geobacter*, which was considered as a typical exoelectrogens in MFCs (Yang et al*.*, 2019) increased from 9.66% (Blank) to 28.01% (C4-HSL) and 24.17% (PQS) during enrichment, and from 3.76% (Blank) to 23.51% (C4-HSL) and 19.17% (PQS) after CTC domestication. On the other hand, *Enterococcus* became the dominant specie in EABs after CTC domestication with the proportion of 47.64% (C4-HSL), 42.96% (PQS), and 38.26% (Blank), respectively. It was reported that *Enterococcus* was the dominant genus for the degradation of tetracycline antibiotics (Zahid et al., 2017). Consequently, under the stress of relative high concentration of CTC, it was firstly considered to degrade CTC to weaken the negative effect on the EABs, which could be explained that the percentage of *Enterococcus* increased after CTC addition. Undoubtedly, we found that the proportion of *Enterococcus* in QS signals groups was higher than that of the Blank group, which could be the result from the positive regulation on EABs by QS signals to resist the stress of high concentration of CTC. Moreover, *Lactococcus* and *Enterococcus* belong to Gram-positive bacteria while *Geobacter* belongs to Gram-negative bacteria. C4-HSL and PQS used in this study were reported to play an important role in the QS systems of Gram-negative bacteria (Brindhadevi et al., 2020; Schuster et al., 2013). Therefore, the proportion of *Geobacter* increased during EABs maturation in the QS group of inoculated MFCs, which resulted in a relative decline in the proportion of *Lactococcus* and *Enterococcus*. Furthermore, the microbial composition of EABs was accordingly altered by the dual regulation of CTC and QS signals to adapt to the change of complex external environment due to the addition of CTC. This dynamic shift was consistent with the variable voltage output and the change in diversity and abundance indices (Li et al., 2019a). Although the two QS signals (C4-HSL and PQS) were mainly reported to affect Gram-negative microorganism rather than Gram-positive microorganism, we could not exclude that the microorganism regulated by QS signals attempted to communicate with other functional species (such as CTC degradative bacteria) to optimize a better survival environment. Generally speaking, fermentative bacteria gradually decreased with the addition of QS signals, while that of exoelectrogens and CTC-degrading bacteria increased, which varied the performance of the EABs confirmed by the above analyses.

The results also indicated that the evolution of EABs varied greatly under the external environment pressure (CTC tolerance in this study), which may be attributed to the different responses and adaptabilities to the QS signals among different microorganisms species. To be more specific, the proportion of exoelectrogens significantly increased while that of fermentative bacteria decreased due to that the QS signals promoted the proportion of CTC-degrading species to reduce the toxicity of CTC and alleviated the unfavorable effects on the exoelectrogens simultaneously.

### Conclusion

In this study, we revealed that QS signals accelerated the evolution of EABs, shortened the start-up time, and increased the voltage output of MFCs systems. The percentage of exoelectrogens was significantly increased under the regulation of QS signals, which reduced the charge transfer resistance of the EABs, and thus enhanced EET within the interface between EABs and anodes. Under the tolerance of CTC, the QS signals further increased the proportion of *Enterococcus* for the efficient degradation of CTC, which moderated the stress of CTC on the performance of exoelectrogens by the positive synergistic effect among mixed species embedded in EABs. These results indicated that QS signals provided a feasible strategy for improving the performance of bioelectrochemical systems (BESs) in electricity production and the treatment of wastewater for sustainable bioremediation.

### Limitations of the study

Here, we concluded that QS signals induced the heterogeneous distribution of EPS to active response for a good balance between self-protection and EET under environmental pressure. The scope of this study is limited to the bench-scale bioelectrochemical systems. We investigated the effects of QS signals (10 μmol/L) on power output, chlortetracycline degradation, and structure of EABs in MFCs treating wastewater containing CTC. Whether this concentration could exhibit the best effect in the mix culture system for pilot-scale bioelectrochemical systems is deserved further study. Besides, the detail understanding of the EPS composition with several electrochemical active substrates in EABs could help us to further understand the effects of QS signals on extracellular electron transfer, which was also limited in this study. In sum, the results provided a broader understanding on the regulation of EABs within electrode interface to improve their performance for environmental remediation and clean energy development.

## STAR★Methods

### Key resources table

**Table:** 

REAGENT or RESOURCE	SOURCE	IDENTIFIER
**Chemicals**		

Glucose	Macklin	Cat# G6172
Na_2_HPO_4_	Macklin	Cat# S818103
NaH_2_PO_4_	Macklin	Cat# M888173
NH_4_Cl	Rhawn	Cat# R009612
KCl	Macklin	Cat# P816347
C4-HSL	Rhawn	Cat# R061799
PQS	Glpbio	Cat# GC45912
EDTA	Macklin	Cat# E809068
CTC	Macklin	Cat# C822258
Potassium ferricyanide	Rhawn	Cat# R018646
Oxalic acid	Macklin	Cat# O815177
Acetonitrile	Macklin	Cat# A800362

**Others**		

Electrochemical workstation	Metrohm	PGSTAT302N
Proton exchange membrane	Dupont	Nafion-117
High Performance Liquid Chromatography	Agilent	1260LC
Scanning electron microscopy	Hitachi	N/A
Confocal laser scanning microscope	LEICA	N/A

### Resource availability

#### Lead contact

Further information and requests for resources and reagents should be directed to and will be fulfilled by the lead contact, Xiao-Yu Yong (yongxiaoyu@njtech.edu.cn).

#### Materials availability

This study did not generate new unique reagents.

### Experimental model and subject details

Our study does not use experimental models typical in the life sciences.

### Methods details

#### Enrichment and gradient domestication of EABs

A dual-chamber MFC with 30 mL effective volume was used in this study which was separated by a proton exchange membrane (Nafion-117, Dupont, 3.0 × 3.0 cm). Two pieces of carbon felt (2 × 2 cm) were used as both anode and cathode, respectively, and connected with an external resistance of 1,000 Ω (titanium wire, φ = 0.5 mm) to form a closed circuit. The anolyte (g/L) was consisted of glucose (2.00), Na_2_HPO_4_ (4.0896), NaH_2_PO_4_ (2.544), NH_4_Cl (0.31), KCl (0.13), minerals 12.5 (mL/L), vitamins 5 (mL/L). The catholyte contained potassium ferricyanide (50 mmol/L) and phosphate buffer (pH 7.0). All reactors were placed in an incubator at 30°C during the whole experiment.

The inoculum (activated sludge), which was sampled from Nanjing wastewater treatment plant (Nanjing, China), was inoculated into the MFCs anode chamber. The sludge and the anolyte were mixed in a ratio of 2:1 (v/v), and 30 mL mixture was inoculated into the anode chamber, which was deoxygenated by bubbling N_2_ for 20 min before use (Li et al., 2019a). QS signals namely C4-HSL and PQS with a final concentration of 10 μmol/L were added to each anode chambers (Chen et al., 2017b). Both of the experimental group and the blank group (without QS signals) were set in triplicates. When the voltage output drops to 20 mV, the anolyte and catholyte was replaced.

To investigate the influence of CTC on the measured EABs, CTC at four incremental concentrations (5, 10, 20 and 30 mg/L) were gradually added to the anode chambers for the further acclimation of EABs. The fermentative groups were the same as the MFC anode chambers while with an open circuit.

#### Electrochemical testing

Cyclic voltammetry (CV) was performed in three-electrode system using an electrochemical workstation (PGSTAT302N, Metrohm) for better understanding about the redox ability of the bioanodes and EET between the anode and EABs. The anode (carbon felt), cathode (carbon felt), and Ag/AgCl electrode were employed as the working electrode, counting electrode, and reference electrode, respectively (Izadi et al., 2020). Different sweep speeds (0.01, 0.02, 0.05, 0.07, 0.08, 0.10 V/s) were selected with a sweep interval of −0.8 to 0.8 V. According to Laviron theory (Laviron and Roullier, 1980), the apparent electron transfer rate was calculated as a linear function of the logarithm of the potential of the redox peaks at different scanning speeds.

The polarization and power density curves of the MFC were measured by varying the external resistance (600-10,000Ω). The current density (*I=U/RA*) and power density (*P=UI*) were calculated from the electrode surface area (4.0 cm^2^), where U is the cell voltage (V), R is the external resistance value, and A is the electrode surface area (Li et al*.*, 2021).

The electrochemical impedance spectroscopy (EIS) was employed in three-electrode system, which was same to the CV experiment, to obtain the ohmic and charge transfer resistances (Izadi et al*.*, 2020), and the results were fitted using ZSim-demo software.

The inhibition rate of different concentrations of CTC on the voltage output of related MFCs in four batches.

$$\text{Inhibition }\left(\text{\%}\right)\;=\;\frac{V\max-V}{Vmax}\times 100\%$$

where Vmax is the maximum output voltage for each group of MFCs during enrichment, V is the maximum voltage for different batches at different concentrations of CTC domestication.

#### Morphological characteristics, activity, and community analysis of EABs

The microscopic morphology of EABs at the early (first cycle), middle (fourth cycle), and late (eighth cycle) stages during enrichment were observed by scanning electron microscopy (SEM). The biomass of EABs was measured by the BCA protein kit (Thermo, USA). Each group of EABs was stained with Live/Dead kit (L7007, Thermo Fisher Scientific Inc, USA), and then the bioactivity and 3D structure of EAB were observed with a laser confocal microscope (CLSM, LEICA DM6000B, Germany).

To characterize changes in the microbial community before and after the acclimation of CTC, microbial community analysis of EABs were carried out according to previous study (Li et al*.*, 2021). Briefly, total DNA was extracted using the PowerSoil DNA isolation kit (Mo-Bio, Carlsbad, CA, USA), and the V4 region of the 16s rRNA gene were amplified using bacterial primers 338F (5′-ACTCCTACGGGAGGCAGCA-3′) and 806R (5′-GGACTACHVGGGTWTCTAAT-3′) (Illumina PE300, Majorbio, Shanghai, China). Microbial composition was analyzed on the Majorbio CloudPlatform (www.Majorbio.com).

#### EPS extraction and measurement

The EPS from the EABs in the late stage of biofilm enrichment was extracted and collected following a method described previously. In brief, the carbon felt in the anode chamber was cut and placed in an EP tube containing 30 mL of pure water, followed by adding 2% EDTA (5 mL) and diluting to 50 mL with pure water. The mixture were shaken and extracted for 5 h at 20°C, and then centrifuged at 14,000 rpm for 20 min. Finally, the supernatant was filtered through a filter membrane to obtain EPS. The Polysaccharide and protein concentrations were measured using the sulfuric acid-phenol method and BCA protein Assay Kit (Thermo, USA) (Li et al., 2021), respectively.

#### CTC degradation curve and kinetic curve

CTC (20 mg/L) was added to the MFC anode chamber after gradient acclimation, and the concentration was detected by high performance liquid chromatography every 2 h. A CTC degradation kinetic curve was established according to the following equation:

(Equation 1) LN(C0/Ct)=Kt

where C0 is the initial CTC concentration (20 mg/L), Ct is the CTC concentration at time t, and K is the first-order kinetic rate coefficient, t is sampling time.

### Quantification and statistical analysis

Figures represent averaged or representative results of multiple independent experiments. Analyses and plots were performed with Origin.

## Data Availability

• All data reported in this paper will be shared by the lead contact upon request. • This paper does not report any original code. • Any additional information required to reanalyze the data reported in this paper is available from the lead contact upon request.
