# Supplementary material for: Quorum sensing signals improve the power performance and chlortetracycline degradation efficiency of mixed-culture electroactive biofilms
Source: iScience. 2022 Apr 26;25(5):104299. doi: 10.1016/j.isci.2022.104299 (PMC9097700; doi:10.1016/j.isci.2022.104299)
Supplement: Document S1. Figures S1–S6 and Tables S1–S3 [file mmc1.pdf]

iScience, Volume *25*

Supplemental information

Quorum sensing signals improve the power performance and chlortetracycline degradation efficiency of mixed-culture electroactive biofilms

Xiao-Long Cheng, Qiang Xu, Jia-Dong Sun, Chun-Rui Li, Qian-Wen Yang, Biao Li, Xue-Ying Zhang, Jun Zhou, and Xiao-Yu Yong

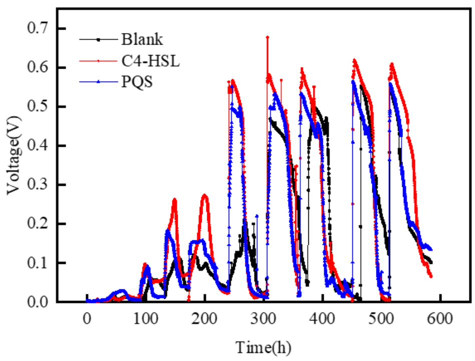

Fig. S1. The voltage output of three MFCs groups during enrichment. Related to Figure 2.

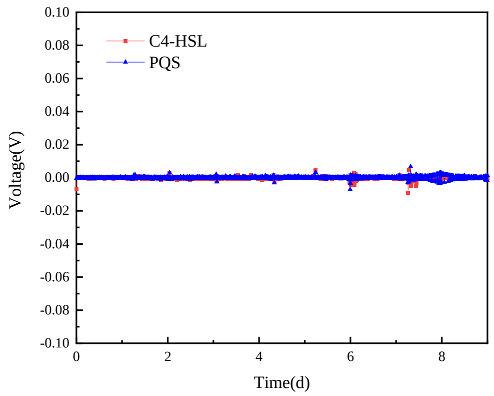

Fig. S2. The voltage output of abiotic groups with C4-HSL and PQS. Related to STAR Methods.

.

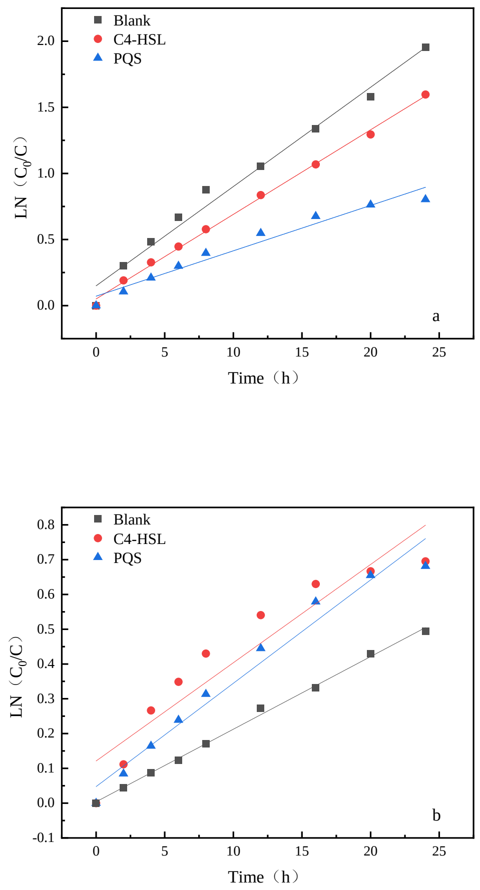

Fig. S3. Chlortetracycline degradation kinetic curve of three MFCs groups; a) degradation in MFCs; b) fermentative degradation. Related to Figure 3.

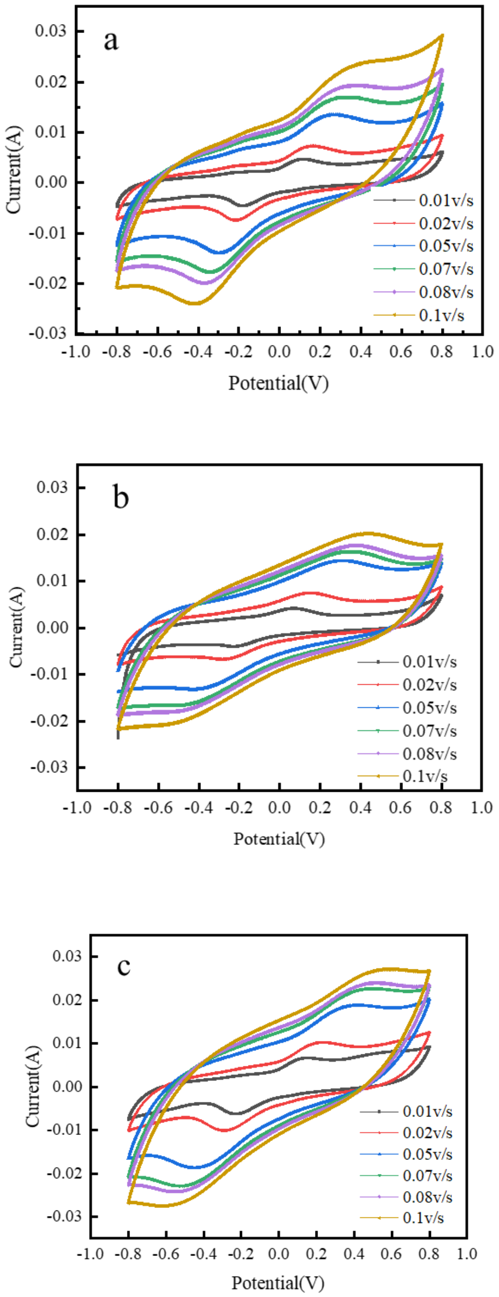

Fig. S4. Cyclic voltammograms of three MFCs groups at various scan rates. a) Blank group; b) C4-HSL group; c) PQS group. Related to STAR Methods.

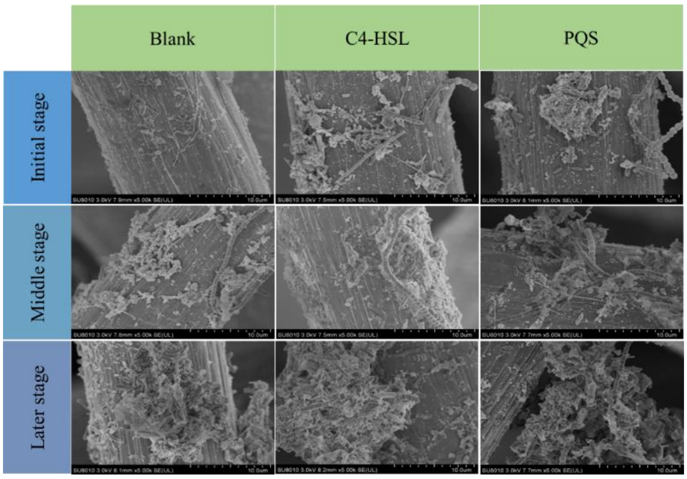

Fig. S5. SEM images collected for the Blank, C4-HSL and PQS groups in the initial, middle and later stages. Related to STAR Methods.

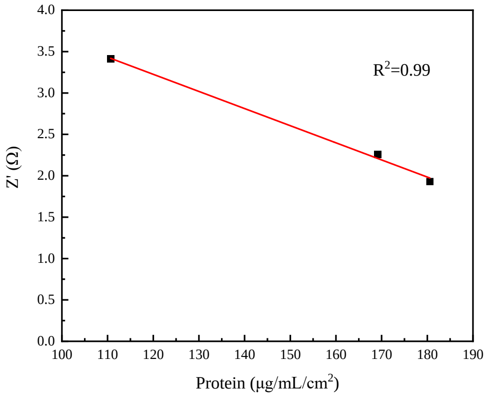

Fig. S6. Correlation between Rct and the protein content in the EPS. Related to Figure 4 and Figure 5.

Table. S1. The partial Information of QS signals (N-butyryl homoserine lactone, C4-HSL; 2-heptyl-3-hydroxy-4-quinolone, PQS) and chlortetracycline (CTC) used in this study. Related to STAR Methods.

Name	CAS	Molecular Formula	Molecular structure
C4-HSL	67605-85-0	$C_8H_{13}NO_3$	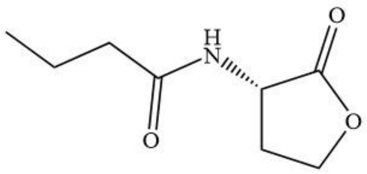
PQS	108985-27-9	$C_{16}H_{21}NO_2$	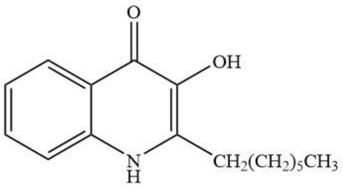
CTC	57-62-5	$C_{22}H_{23}ClN_2O_8$	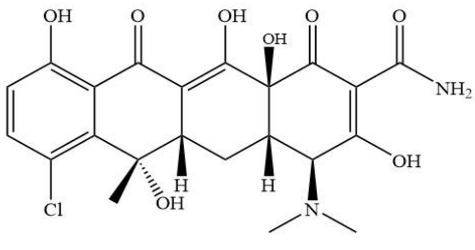

Table. S2. The concentration of CTC added in the anode chamber for the further domestication of EABs. Related to STAR Methods.

**Stages**	**Cycles**	**Substrate**	
		Individual CTC (mg/L)	Glucose (mg/L)
I	1	5	2000
	2		1800
	3		1600
	4		1400
	5		1200
	6		1000
II	1-6	10	1000
III	1	20	1000
	2		800
	3		600
	4		400
	5		200
	6		0
IV	1-6	30	0

Table. S3. The degradation rate constant of CTC in three groups. Related to Figure 3.

	C4-HSL	PQS	Blank
Degradation in MFCs	0.07511±0.003	0.06398±0.001	0.03434±0.002
R^2	0.9844	0.9977	0.9633
Fermentative degradation	0.02825±0.0036	0.02971±0.0019	0.02087±0.0004
R^2	0.8964	0.9703	0.9976
